# Game theory, learning, and control systems

**DOI:** 10.1093/nsr/nwz163

**Published:** 2019-11-04

**Authors:** Jeff S Shamma

**Affiliations:** Computer, Electrical and Mathematical Science and Engineering Division, King Abdullah University of Science and Technology, Saudi Arabia

## Abstract

Game theory is the study of interacting decision makers, whereas control systems involve the design of intelligent decision-making devices. When many control systems are interconnected, the result can be viewed through the lens of game theory. This article discusses both long standing connections between these fields as well as new connections stemming from emerging applications.

Game theory is the study of interacting decision makers [1], i.e. settings in which the quality of an actor’s decision depends on the decisions of others. In commuting, the congestion experienced on a road depends on a vehicle’s path, as well as the paths taken by other vehicles. In auctions, the outcome depends on one’s own bid as well as the bids of others. In competitive markets, market share depends on both a firm’s pricing as well as the pricing of its competitors.

While game theory traditionally has been studied within the realm of mathematical social sciences, there are also strong ties to control systems [2,3].

A longstanding connection is the setting of zero-sum, or minimax, games. In zero-sum games, there are two players, and a benefit to one player is a detriment to the other. A classical example is pursuit-evasion games [4]. A common perspective in control systems is that one player is the controller and the opposing player is an adversarial environment, e.g. exogenous disturbances [5] or model misspecification [6]. The controller seeks to optimize a specified performance objective, whereas the adversarial environment seeks to reduce achieved performance. There has been renewed interest in zero-sum games in the area of security [7,8], where security measures are to be taken against a variety of adversarial attacks ranging from intrusion to data corruption to privacy violation.

Another area of interest is in distributed or networked control systems [9], motivated by applications such as power networks, transportation networks, and multi-robot systems. In such settings, illustrated in Fig. 1, there is a large number of decision making components where no single actor has full information on the state of the environment or full authority over the decisions over the network. A representative application is the smart grid [10], where a distributed network of prosumers make decisions on production, consumption, and storage of energy in response to evolving demand and environmental conditions.

**Figure 1.**
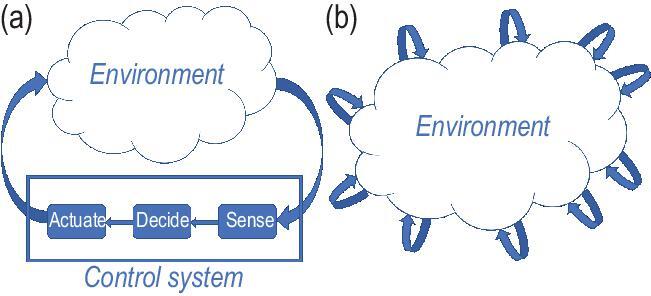
(a) A traditional control system architecture with centralized information and authority. (b) A distributed or networked control system architecture with multiple interacting decision makers.

A more recent connection between game theory and control systems is in the area of game-theoretic learning [11,12].

To set up the discussion, we first define a (non-cooperative) game by (i) a set of players; (ii) for each player, a set of actions; and (iii) for each player, a utility function that quantifies a player’s satisfaction with the collective actions of all players. More formally, we can write the utility function of the *i*th player as *U*_*i*_(*a*_1_, *a*_2_, $\ldots$, *a*_*i*_, $\ldots$, *a*_*n*_), where (*a*_1_, $\ldots$, *a*_*n*_) is the action profile of *n* players, *a*_*i*_ is the action of the *i*th player, and *U*_*i*_(·) is a real-valued function where the *i*th player prefers the action profile *a* = (*a*_1_, $\ldots$, *a*_*n*_) over $a^{\prime } = (a_1^{\prime },\ldots ,a_n^{\prime })$ whenever *U*_*i*_(*a*) > *U*_*i*_(*a*^′^) (i.e. larger utility is better).

An important concept in game theory is the Nash equilibrium, which is an action profile $(a_1^*,\ldots ,a_n^*)$ such that for any player *i* and every alternative action $a_i^{\prime }$, i.e. each player’s action is optimal with respect to the actions of other players.


\begin{eqnarray*} U_i(a_1^*,\ldots ,a_i^*,\ldots ,a_n^*)\\ \ge U_i(a_1^*,\ldots ,a_i^{\prime },\ldots ,a_n^*), \end{eqnarray*}

Nash equilibrium is an example of a solution concept for a game, which is a proposed outcome given the specification of the elements of a game. Such an interpretation can be problematic. A game may have multiple Nash equilibria, resulting in an issue of non-uniqueness. Another lingering question is how agents might reach a Nash equilibrium, especially given that agents have limited knowledge about the utility functions of other agents or even observations of the actions of other agents. Indeed, even computing a Nash equilibrium can have intractable computational complexity [13]. Nonetheless, Nash equilibrium is widely used as representative of the outcome of a game-theoretic model.

The study of game-theoretic learning partially addresses these issues by shifting the discussion away from Nash equilibrium and towards how players might reach a Nash equilibrium through some sort of online or adaptive learning process. Such learning processes evolve over stages, e.g. *t* = 0, 1, 2, $\ldots$, and can be represented as

\begin{eqnarray*} a_i(t) \sim \mathcal {LR}[\mathcal {I}_i(t); U_i(\cdot )], \end{eqnarray*}

where the action of the *i*th player at stage *t* is determined by the learning rule, $\mathcal {LR}[\cdot ]$, that acts on the information available to player *i* up to stage *t*, $\mathcal {I}_i(t)$, as well as *U*_*i*_(·), the specific utility function of player *i*. A learning rule may be stochastic, wherein the action is a randomized outcome according to a probability distribution generated by the learning rule. For example, in reinforcement learning, an action is selected with a probability that is proportional to the cumulative utility that it has garnered in the past.

There is a very interesting and complicating factor that distinguishes game-theoretic learning from other learning formulations such as reinforcement learning. An implicit assumption in learning is that there is a stationary environment, and so, over time, one can determine what actions are more effective. However, in game-theoretic learning, the environment comprises other learning agents. Learning in the presence of other learners results in a non-stationary environment from the perspective of any individual agent. Indeed, depending on both the structure of the underlying game and the specific learning rule, outcomes can range from convergence to a Nash equilibrium (or other solution concepts, most notably correlated equilibrium [12]) to preferential selection of some Nash equilibria over others to non-convergence and even chaotic behavior. This notion of embedding learning agents in a common environment recently has gained significant attention in the context of training neural networks through so-called generative adversarial networks [14].

The game-theoretic learning framework leads to two significant connections to control systems. First, game-theoretic learning offers an approach to designing distributed control systems [9], as illustrated in Fig. 1, where the components are programmable engineered devices. An example is in area coverage problems [15], where mobile sensors are to explore an unknown environment. The main idea is to view each component as a player in a game. The system designer must endow these artificial players with both incentives (i.e. utility functions) and adaptive control laws (i.e. learning rules) that induce a desirable collective behavior through local interactions that respect the underlying distributed decision architecture.

A second connection is that control theory offers new insights into the analysis of game-theoretic learning. A learning rule is a type of dynamical system, and so interacting learning agents constitute a feedback interconnection of dynamical systems with special structures emerging from game-theoretic learning. Recent work includes exploiting underlying passivity properties associated with game-theoretic learning rules (see the tutorial paper [16] and references therein).

These research directions represent complementary paradigms for game-theoretic learning. In the first, game-theoretic learning is being used as a prescriptive approach to programming engineered devices. In the second, game-theoretic learning is a descriptive approach to modeling evolving human decision making. Going forward, there is a significant opportunity for game theory and control systems that blend these two perspectives.

In the emerging area of cyber–physical–social systems, the distributed decision architecture in Fig. 1 is a possible mix of both programmable devices and human decision makers. An application area is in smart cities [17], where (i) human drivers may share the road with autonomous vehicles; (ii) human users must be incentivized into participating in energy demand response while monitored by IoT devices; and (iii) humans and robots interact in unstructured environments, such as in assistive robotics. In such applications, the perspectives of both game theory and control theory come together, where game theory models interactive decision making and control systems methods address evolutionary dynamics while mitigating the uncertainty inherent in human decision making.
